# Association between cardiopulmonary bypass duration and early major adverse cardiovascular events after surgical repair of supravalvular aortic stenosis

**DOI:** 10.3389/fcvm.2025.1519251

**Published:** 2025-01-21

**Authors:** Simeng Zhang, Caiyi Wei, Bo Peng, Lizhi Lv, Fengbo Pei, Jianming Xia, Jun Yan, Jie Liu, Qiang Wang, Yi Shi

**Affiliations:** ^1^ Department of Cardiac Surgery, Peking University People’s Hospital, Beijing, China; ^2^ School of Basic Medical Sciences, Peking University, Beijing, China; ^3^ Department of Cardiac Surgery, National Center for Cardiovascular Diseases and Fuwai Hospital, Chinese Academy of Medical Sciences & Peking Union Medical College, Beijing, China; ^4^ Department of Pediatric Cardiac Surgery, Beijing Anzhen Hospital, Capital Medical University, Beijing, China; ^5^ Department of Cardiac Surgery, Yunnan Fuwai Cardiovascular Hospital, Kunming, China; ^6^ Department of Vascular and Endovascular Surgery, Chinese PLA General Hospital, Beijing, China

**Keywords:** cardiopulmonary bypass, supravalvular aortic stenosis, adverse cardiovascular events, surgical therapy, logistic regression analysis

## Abstract

**Background:**

Patients who underwent surgical repair of supravalvular aortic stenosis (SVAS) are at high risk for postoperative major adverse cardiovascular events (MACE). This study aimed to investigate the association between cardiopulmonary bypass (CPB) duration and MACE occurring during postoperative hospitalization or within 30 days post-surgery.

**Methods:**

Patients who underwent surgical repair of SVAS from 2002 to 2019 at Beijing Fuwai Hospital and Yunnan Fuwai Hospital were included in this study. Patients were stratified into “CPB duration >2 h” and “CPB duration ≤2 h” groups based on intraoperative CPB duration. Various statistical methodologies were employed to investigate the association between CPB duration and early postoperative MACE, including multivariate adjustment, propensity score adjustment, propensity score matching, and logistic regression based on propensity score weighting.

**Results:**

297 participants were included and 164 were finally matched. In the propensity score-matched cohort, CPB duration was positively associated with early postoperative MACE (odds ratio = 18.13; 95% confidence interval 2.33–140.86; *P* = 0.006). Consistent results were obtained in the Inverse probability of treatment-weighted, standardized mortality ratio-weighted, pairwise algorithmic-weighted, and overlap-weighted models.

**Conclusion:**

Patients with CPB duration >2 h were at a higher risk of early postoperative MACE compared to those with CPB duration ≤2 h. This emphasized the significance of minimizing CPB exposure for the prognosis of patients with SVAS.

## Introduction

Supravalvular aortic stenosis (SVAS) is a rare form of congenital outflow tract obstruction with an incidence of 1/20,000 births (1). Pulmonary artery stenosis is associated with SVAS, and the incidence of SVAS has been reported to range from 45% to 75% in patients with Williams–Beuren syndrome (WBS) (2). Two types of SVAS are typically seen: a discrete narrowing typically located at the sinotubular junction or a diffuse obstruction of the ascending aorta and even involving the descending aorta (2, 3).

The natural course of SVAS is progressive and the majority of the patients require surgical interventions (4). Most performed surgical techniques for SVAS correction include: McGoon repair, Doty repair, Brom repair, and Myers sliding aortoplasty. These techniques have relatively low mortality (5–9). However, the rates of reintervention and aortic insufficiency vary across different surgical techniques (4). Specifically, the duration of cardiopulmonary bypass (CPB) is different between some surgical techniques (4, 5). Previous studies have primarily focused on the differences in outcomes among various surgical procedures. Nonetheless, the effect of CPB duration on early major adverse cardiovascular events (MACE) following surgical repair of SVAS is poorly understood. No study to date has utilized propensity score matching (PSM) to investigate the risk factors for MACE following surgical repair of SVAS.

CPB refers to the use of a pump-oxygenator device to partially or completely replace the functions of the heart and lung, usually for performing safe cardiac operations (10). Notably, the use of CPB has been recognized as an independent predictor of postoperative mortality in patients undergoing cardiac surgery (11, 12). The CPB-induced proinflammatory response, organ dysfunction, and sublingual microcirculatory perfusion contribute to postoperative morbidity and mortality (13, 14). However, few studies have reported the relationship between CPB used during a specific procedure (other than coronary artery bypass grafting) and postoperative cardiovascular adverse events. Accordingly, this study employed propensity score matching analyses to identify the association between CPB duration and early MACE.

## Materials and methods

### Patient population

**Figure 1:**
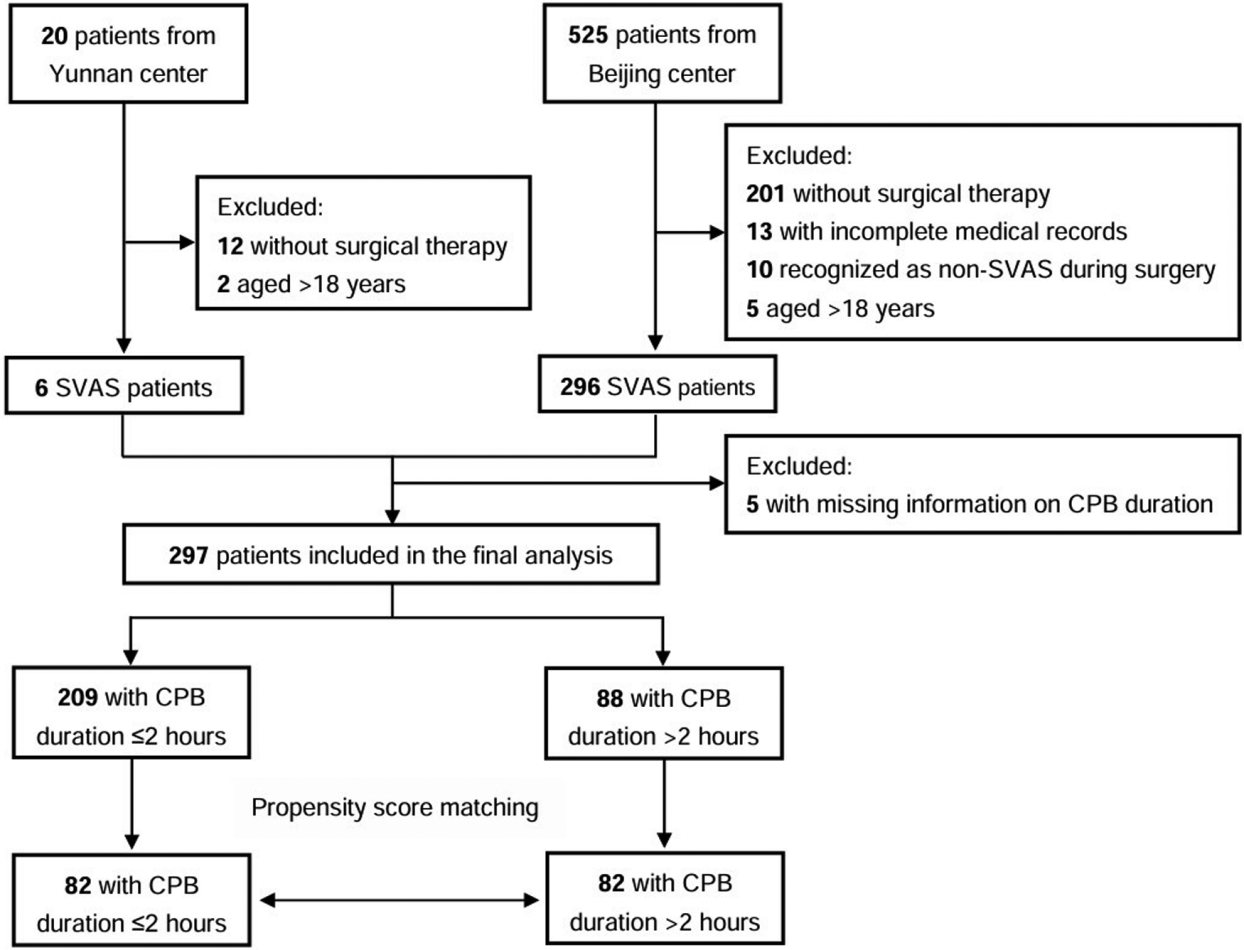
Study flow chart; CPB, cardiopulmonary bypass.

This study evaluated medical records of 575 patients with SVAS who underwent surgical therapy at Beijing Fuwai Hospital and Yunnan Fuwai Hospital between 2002 and 2019. The surgical techniques included McGoon repair, Doty repair, Brom repair, Myers sliding aortoplasty, and others. The inclusion criteria for patients were as follows: (1) age <18 years, (2) diagnosis of SVAS based on echocardiography or intraoperative findings, and (3) undergoing first-time surgical repair of SVAS with the use of CPB (Figure 1).

### Baseline demographic and clinical characteristics

Baseline characteristics included sex, age, weight, height, body surface area, cardiothoracic ratio, arrhythmia, left ventricular (LV) hypertrophy, WBS, cardiac- and aortic-surgery, and form of SVAS. Preoperative echocardiographic variables were included, such as ejection fraction (EF), supravalvular aortic blood velocity, gradients of the stenosis, transvalvular gradients, LV end-diastolic diameter and systolic diameter, aortic annulus diameter, aortic sinus diameter, stenosis diameter, diameter of ascending aorta. Concomitant malformation variables were included, such as tetralogy of Fallot, aortic arch hypoplasia, hypertrophic obstructive cardiomyopathy, bicuspid aortic valve, mitral valve regurgitation, aortic valve regurgitation, stenosis of pulmonary artery, pulmonary valve, mitral valve, and aortic valve.

### Outcomes

The primary endpoint included surgery-related mortality due to surgical complications or other diseases, heart- or aorta-related complications, and the need for reintervention.

MACE was defined as events occurring during postoperative hospitalization or within 30 days post-surgery, including malignant arrhythmias, cardiac arrest, extracorporeal membrane oxygenation (ECMO) needed, reoperation, or in-hospital mortality. Malignant arrhythmias were defined as arrhythmias that cause hemodynamic disorders in patients within a short period, leading to syncope or even sudden death, including ventricular fibrillation, ventricular tachycardia, and multiform premature ventricular contractions. Restenosis at follow-up was defined as a mean supravalvular aortic gradient >40 mmHg (15–17).

### Data analysis

Continuous variables were reported as mean ± standard deviation (SD) and compared using independent samples *t*-test. Categorical variables were reported as frequencies and percentages, and the group difference was tested by χ^2^ test or Fisher's exact test. *Z*-score was calculated using the Boston Children's Hospital Echocardiography Calculation Tool (https://zscore.chboston.org/) (accessed on November 22, 2021).

Participants were categorized into two groups based on CPB duration: “CPB duration >2 h” and “CPB duration ≤2 h”. To minimize allocation bias and confounding, a 1:1 nearest neighbor propensity score-matched cohort was constructed. Using the estimated propensity scores (PS) as weights, the inverse probability of treatment-weighted (IPTW), standardized mortality ratio-weighted (SMRW), pairwise algorithmic-weighted (PA), and overlap-weighted (OW) models were used to generate a weighted cohort.

To investigate the association between CPB duration and MACE, multivariate-adjusted logistic regression models were performed based on the full cohort, the PS-matched cohort and the weighted cohorts. To validate the robustness of the findings, an additional multivariate-adjusted logistic regression model was performed on the full cohort, including the propensity score as a covariate in the analysis. The results were reported as *P*-values, odds ratios (OR), and 95% confidence intervals (CI). Statistical significance was defined as a two-sided *P*-value of <0.05. R software (version 4.0.3) and Free Statistics software (version 1.6) were used for all the statistical analyses.

## Results

### Baseline characteristics

Out of 297 patients enrolled in this study, 88 (29.6%) experienced CPB for more than 2 h (Table 1). Before PSM, several baseline characteristics between “CPB duration ≤2 h” and “CPB duration >2 h” groups were significantly different. For instance, compared to patients with CPB duration ≤2 h, patients with CPB duration >2 h had higher age at operation (150.89 ± 170.19 vs. 99.56 ± 116.04, *P* = 0.003), greater weight (31.25 ± 24.90 vs. 25.78 ± 20.15, *P* = 0.048), larger cardiothoracic ratio (0.54 ± 0.09 vs. 0.50 ± 0.06, *P* < 0.001), wider LV end-diastolic diameter (37.37 ± 14.50 vs. 33.28 ± 6.98, *P* = 0.002), and wider aortic annulus diameter (15.04 ± 4.62 vs. 14.00 ± 3.43, *P* = 0.039). Moreover, the incidence of mitral stenosis (5.68% vs. 0%, *P* = 0.002), mitral valve regurgitation (15.91% vs. 7.18%, *P* = 0.021), aortic valve stenosis (28.41% vs. 17.7%, *P* = 0.038) and aortic valve regurgitation (18.18% vs. 9.09%, *P* = 0.027) were higher in the “CPB duration >2 h” group. Additionally, patients with CPB duration ≤2 h exhibited higher rates of discrete SVAS (96.17% vs. 89.77%, *P* = 0.030) and higher EF (70.48 ± 6.32 vs. 66.66 ± 10.41, *P* < 0.001).

**Table 1:** Baseline characteristics of supravalvular aortic stenosis patients before propensity score matching.

Variables	Total (*n* = 297)	CPB duration ≤2 h (*n* = 209)	CPB duration >2 h (*n* = 88)	*P*
Sex, *n* (%)				
Male	196 (65.99)	141 (67.46)	55 (62.5)	0.41
Female	101 (34.01)	68 (32.54)	33 (37.5)	
Age at operation, m	114.77 ± 136.11	99.56 ± 116.04	150.89 ± 170.19	**0.003**
Weight, kg	27.40 ± 21.77	25.78 ± 20.15	31.25 ± 24.90	**0.048**
Height, cm	113.32 ± 33.72	111.68 ± 31.15	117.22 ± 39.08	0.199
BSA, m^2^	0.89 ± 0.47	0.86 ± 0.43	0.96 ± 0.55	0.09
Cardiothoracic ratio	0.51 ± 0.07	0.50 ± 0.06	0.54 ± 0.09	**<0.001**
Arrhythmia	23 (7.74)	17 (8.13)	6 (6.82)	0.698
LVH	147 (50.00)	102 (49.04)	45 (52.33)	0.608
Williams-Beuren syndrome	23 (7.74)	16 (7.66)	7 (7.95)	0.93
Cardiac surgery	3 (1.01)	3 (1.44)	0 (0)	0.557
Aortic surgery	7 (2.36)	3 (1.44)	4 (4.55)	0.202
Type of SVAS				
Discrete	280 (94.28)	201 (96.17)	79 (89.77)	**0.030**
Diffuse	17 (5.72)	8 (3.83)	9 (10.23)	
Cardiac echocardiography				
EF	69.37 ± 7.92	70.48 ± 6.32	66.66 ± 10.41	**<0.001**
Supra-aortic blood velocity, m/s	4.31 ± 1.11	4.24 ± 1.06	4.47 ± 1.21	0.126
Gradients of the stenosis, mmHg	78.91 ± 34.42	75.89 ± 31.41	85.66 ± 39.80	0.065
Transvalvular gradients, mmHg	75.70 ± 36.57	73.33 ± 34.48	81.24 ± 40.75	0.112
LV end-diastolic diameter, mm	34.49 ± 9.98	33.28 ± 6.98	37.37 ± 14.50	**0.002**
LV end-systolic diameter, mm	20.94 ± 9.32	20.40 ± 8.78	22.67 ± 11.01	0.414
Aortic annulus diameter, mm	14.31 ± 3.84	14.00 ± 3.43	15.04 ± 4.62	**0.039**
Aortic annulus *z*-score	0.11 ± 1.34	−0.01 ± 1.29	0.39 ± 1.43	**0.032**
Aortic sinus inner diameter, mm	18.55 ± 5.69	18.13 ± 5.07	19.56 ± 6.87	0.055
Aortic root *z*-score	1.87 ± 1.77	1.77 ± 1.61	2.12 ± 2.11	0.150
Diameter of the stenosis, mm	8.28 ± 3.10	8.16 ± 2.76	8.58 ± 3.88	0.321
Diameter of ascending aorta, mm	16.22 ± 7.41	15.71 ± 6.42	17.51 ± 9.37	0.072
Ascending aorta *z*-score	−0.64 ± 2.22	−0.75 ± 2.02	−0.35 ± 2.69	0.199
Concomitant malformation				
PAS	54 (18.18)	32 (15.31)	22 (25)	**0.048**
PDA	17 (5.72)	11 (5.26)	6 (6.82)	0.598
VSD	17 (5.72)	9 (4.31)	8 (9.09)	0.105
ASD	21 (7.07)	14 (6.7)	7 (7.95)	0.700
TOF	1 (0.34)	0 (0)	1 (1.14)	0.296
Abnormal coronary artery origin	4 (1.35)	2 (0.96)	2 (2.27)	0.585
Aortic arch hypoplasia	17 (5.72)	10 (4.78)	7 (7.95)	0.283
LVOT stenosis	5 (1.68)	2 (0.96)	3 (3.41)	0.156
HOCM	6 (2.02)	3 (1.44)	3 (3.41)	0.366
PVS	9 (3.03)	6 (2.87)	3 (3.41)	0.727
Bicuspid aortic valve	33 (11.11)	22 (10.53)	11 (12.5)	0.621
Mitral stenosis	5 (1.68)	0 (0)	5 (5.68)	**0.002**
MVR	29 (9.76)	15 (7.18)	14 (15.91)	**0.021**
AVS	62 (20.88)	37 (17.7)	25 (28.41)	**0.038**
AVR	35 (11.78)	19 (9.09)	16 (18.18)	**0.027**
Supravalvular aortic septum	8 (2.69)	3 (1.44)	5 (5.68)	0.053
Subvalvular aortic septum	14 (4.71)	9 (4.31)	5 (5.68)	0.565

CPB, cardiopulmonary bypass; BSA, body surface area; LVH, left ventricular hypertrophy; SVAS, supravalvular aortic stenosis; EF, ejection fraction; PAS, pulmonary aortic stenosis; PDA, patent ductus arteriosus; VSD, ventricular septal defect; ASD, atrial septal defect; TOF, tetralogy of Fallot; LVOT, left ventricular outflow tract; HOCM, hypertrophic obstructive cardiomyopathy; PVS, pulmonary valve stenosis; MVR, mitral valve regurgitation; AVS, aortic valve stenosis; AVR, aortic valve regurgitation.

Level of significance: *P* < 0.05. The bold values indicate statistical significance.

### Propensity score-matched comparison of baseline characteristics of SVAS patients stratified by CPB duration

After PSM, 88 patients with CPB duration >2 h and 88 patients with CPB ≤2 h were matched. Before and after PSM, significant differences were both found in the cardiothoracic ratio, EF, and aortic annulus *Z*-score between the two groups. However, except for the three variables, the discrepancies of almost all baseline variables between the two groups were eliminated, suggesting that there were only minor variations in baseline features between the two groups after properly matching (Table 2).

**Table 2:** Propensity score-matched comparison of baseline characteristics of supravalvular aortic stenosis patients.

Variables	Total (*n* = 164)	CPB duration ≤2 h (*n* = 82)	CPB duration >2 h (*n* = 82)	*P*
Sex				
Male	107 (65.2)	56 (68.3)	51 (62.2)	0.512
Female	57 (34.8)	26 (31.7)	31 (37.8)	
Age, m	132.9 ± 152.5	110.1 ± 124.0	155.6 ± 174.2	0.056
Weight, kg	29.6 ± 22.4	27.0 ± 18.9	32.2 ± 25.4	0.140
Height, cm	117.6 ± 35.3	116.7 ± 30.5	118.4 ± 39.7	0.752
BSA, m^2^	0.9 ± 0.5	0.9 ± 0.4	1.0 ± 0.6	0.249
Cardiothoracic ratio	0.5 ± 0.1	0.5 ± 0.1	0.5 ± 0.1	**0.019**
Arrhythmia	8 (4.9)	3 (3.7)	5 (6.1)	0.720
LVH	81 (49.7)	38 (46.3)	43 (53.1)	0.481
Williams-Beuren syndrome	13 (7.9)	6 (7.3)	7 (8.5)	>0.99
Cardiac surgery	2 (1.2)	2 (2.4)	0 (0)	0.497
Aortic surgery	6 (3.7)	2 (2.4)	4 (4.9)	0.682
Form of SVAS				
Discrete	147 (89.6)	74 (90.2)	73 (89)	>0.99
Diffuse	17 (10.4)	8 (9.8)	9 (11)	
Cardiac echocardiography				
EF	68.7 ± 9.3	71.3 ± 6.2	66.1 ± 11.0	**<0.001**
Supra-aortic blood velocity, m/s	4.4 ± 1.2	4.3 ± 1.2	4.5 ± 1.2	0.390
Gradients of the stenosis, mmHg	81.9 ± 35.7	78.8 ± 31.1	84.7 ± 39.5	0.387
Transvalvular gradients, mmHg	78.6 ± 39.7	75.0 ± 36.0	82.3 ± 43.0	0.273
LV end-diastolic diameter, mm	35.9 ± 12.0	34.4 ± 7.8	37.5 ± 15.0	0.116
LV end-systolic diameter, mm	21.4 ± 8.7	20.2 ± 5.8	22.8 ± 11.4	0.427
Aortic annulus diameter, mm	14.7 ± 4.2	14.2 ± 3.4	15.1 ± 4.8	0.217
Aortic annulus *z*-score	0.0 ± 1.3	−0.2 ± 1.1	0.3 ± 1.4	**0.018**
Aortic sinus inner diameter, mm	19.3 ± 6.3	18.9 ± 5.3	19.7 ± 7.1	0.416
Aortic root *z*-score	1.9 ± 1.8	1.8 ± 1.6	2.0 ± 2.0	0.510
Diameter of the stenosis, mm	8.5 ± 3.5	8.5 ± 3.1	8.5 ± 3.9	0.886
Diameter of ascending aorta, mm	17.0 ± 8.6	16.6 ± 7.4	17.5 ± 9.7	0.499
Ascending aorta *z*-score	−0.6 ± 2.4	−0.7 ± 2.1	−0.5 ± 2.7	0.592
Concomitant malformation				
PAS	34 (20.7)	12 (14.6)	22 (26.8)	0.083
PDA	6 (3.7)	2 (2.4)	4 (4.9)	0.682
VSD	11 (6.7)	4 (4.9)	7 (8.5)	0.532
ASD	12 (7.3)	6 (7.3)	6 (7.3)	>0.99
TOF	1 (0.6)	0 (0)	1 (1.2)	>0.99
Abnormal coronary artery origin	3 (1.8)	1 (1.2)	2 (2.4)	>0.99
Aortic arch hypoplasia	11 (6.7)	5 (6.1)	6 (7.3)	>0.99
LVOT stenosis	3 (1.8)	1 (1.2)	2 (2.4)	>0.99
HOCM, *n* (%)	4 (2.4)	2 (2.4)	2 (2.4)	>0.99
PVS	6 (3.7)	3 (3.7)	3 (3.7)	>0.99
Bicuspid aortic valve	21 (12.8)	13 (15.9)	8 (9.8)	0.350
Mitral stenosis	0 (0.0)	0 (0.0)	0 (0.0)	>0.99
MVR	22 (13.4)	11 (13.4)	11 (13.4)	>0.99
AVS	46 (28.0)	24 (29.3)	22 (26.8)	0.862
AVR	28 (17.1)	13 (15.9)	15 (18.3)	0.836
Supravalvular aortic septum	6 (3.7)	3 (3.7)	3 (3.7)	>0.99
Subvalvular aortic septum	7 (4.3)	4 (4.9)	3 (3.7)	>0.99

CPB, cardiopulmonary bypass; BSA, body surface area; LVH, left ventricular hypertrophy; SVAS, supravalvular aortic stenosis; EF, ejection fraction; PAS, pulmonary aortic stenosis; PDA, patent ductus arteriosus; VSD, ventricular septal defect; ASD, atrial septal defect; TOF, tetralogy of Fallot; LVOT, left ventricular outflow tract; HOCM, hypertrophic obstructive cardiomyopathy; PVS, pulmonary valve stenosis; MVR, mitral valve regurgitation; AVS, aortic valve stenosis; AVR, aortic valve regurgitation.

Level of significance: *P* < 0.05. The bold values indicate statistical significance.

### The association of CPB duration with MACE

**Figure 2:**
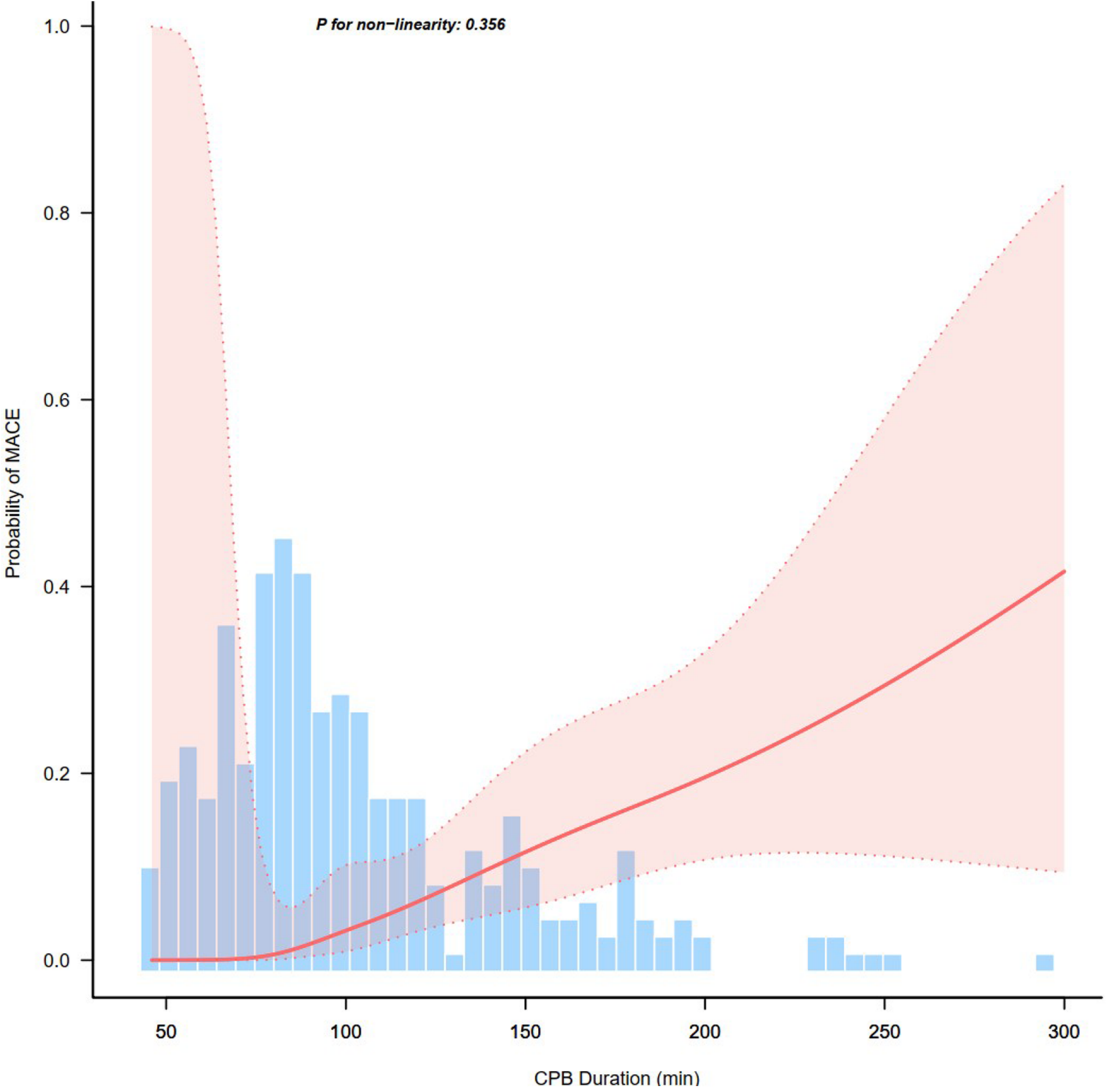
Association between continuous time on cardiopulmonary bypass (CPB) and probability of postoperative major adverse cardiac events (MACE).

Figure 2 showed the effect of continuous time on CPB on the probability of postoperative MACE. Multivariate logistic regression models were performed to determine the association between CPB duration and early postoperative MACE in a series of cohorts (Table 3). In the full cohort, CPB duration was positively associated with MACE (OR = 12.27; 95% CI 4–37.69; *P* < 0.001). The association remained after multivariate adjustment. Patients with CPB duration >2 h had a 11.5-fold greater probability of MACE than those with CPB duration ≤2 h (OR = 12.5; 95% CI 3.93–39.74; *P* < 0.001). Consistently, the OR between CPB duration and MACE was 12.22 (95% CI 3.87–38.56; *P* < 0.001) in PS adjustment and 18.13 (95% CI 2.33–140.86; *P* = 0.006) in PS matching multivariable-adjusted logistic regression model. The results remained robust in the weighted cohort: the IPTW multivariate-adjusted logistic regression model yielded an OR of 13.48 (95% CI 4.48–40.51; *P* < 0.001); the SMRW multivariate-adjusted logistic regression model yielded an OR of 10.53 (95% CI 3.56–31.17; *P* < 0.001); the PA multivariate-adjusted logistic regression model yielded an OR of 9.37 (95% CI 1.95–44.91; *P* = 0.005); the OW multivariate-adjusted logistic regression model yielded an OR of 11.11 (95% CI 1.61–76.74; *P* = 0.015).

**Table 3:** Association between cardiopulmonary bypass duration and MACE in the crude, multivariable, and PS-based analyses.

	OR, 95% CI	*P*-value
Crude analysis	12.27 (4–37.69)	<0.001
Multivariable-adjusted analysis	12.5 (3.93–39.74)	<0.001
Adjusted for propensity score	12.22 (3.87–38.56)	<0.001
With matching	18.13 (2.33–140.86)	0.006
With IPTW	13.48 (4.48–40.51)	<0.001
With SMRW	10.53 (3.56–31.17)	<0.001
With PA	9.37 (1.95–44.91)	0.005
With OW	11.11 (1.61–76.74)	0.015

MACE, major adverse cardiac events; IPTW, inverse probability-of-treatment weighted; SMRW, standardized-mortality-ratio weighted; PA, pairwise algorithmic; OW, overlap weight.

## Discussion

In this multicenter retrospective cohort study, propensity score matching (PSM) analyses revealed that cardiopulmonary bypass (CPB) duration was independently associated with early MACE following surgery repair of SVAS. After PSM, patients with CPB duration >2 h had a 17.13-fold increase in the risk of early postoperative MACE compared to those with CPB duration ≤2 h. After adjusting for the propensity score, the results remained stable. In the IPTW, SMRW, PA, and OW cohorts, CPB duration >2 h was associated with 12.48-, 9.53-, 8.37-, and 10.11-fold increases in the incidence of early postoperative MACE. These results indicated that longer CPB duration linked with worse prognosis of patients with SVAS. By stratifying participants based on intraoperative CPB duration, constructing logistic analyses, and presenting the risk curve, this study provided clinicians a guide for real-time decision making in the operation.

Previous studies have identified risk factors for postoperative mortality and reintervention in patients with SVAS. Diffuse SVAS was a predictor of poor prognosis and reoperation (8, 18, 19). Concomitant aortic valve disease was associated with late mortality (8, 20). Age <1 year at operation was an independent risk factor for early mortality or late death (5, 20). Accordingly, to minimize allocation bias and confounding, our study conducted PSM to balance the baseline characterizes of different groups. After PSM, the differences in age at operation, weight, type of SVAS, and concomitant cardiac diseases (including pulmonary aortic stenosis, mitral stenosis, mitral valve regurgitation, aortic valve stenosis, and aortic valve regurgitation) between the two groups were disappeared.

After PSM, the PS matching multivariable-adjusted logistic regression model still showed that CPB duration was a risk factor of early MACE after surgical repair of SVAS. This implied that our findings were reliable. However, it could not guarantee that all baseline characters were matched. Some potential biases may not be fully eliminated. For instance, CPB duration was found to be significantly different between overall team familiarity terciles (low, medium and high) (21). Although no relationship between cardiothoracic ratio and CPB duration has been reported, future study should further explore other variables independently related to CPB duration and improve the stability of the adjustment model.

With regards to the detrimental effects of prolonged duration of CPB, a multicenter analysis of 216 patients has reported that CPB duration >150 min (OR = 3.5; 95% CI 1.5–8.5; *P* < 0.01) was independently associated with MACE (including the need for postoperative ECMO, CPB, or operative mortality) after repair of truncus arteriosus (22). Patients with MACE had longer duration of CPB (*P* = 0.02) (22). A study analyzing 22,763 patients from the European Congenital Heart Surgeons Association database reported that the use of CPB was associated with a higher mortality rate at 30 days (*P* < .001) and during the hospitalization (*P* < 0.001). Patients receiving ECMO support exhibited longer CPB time (*P* < .001) (12). Consistently our results indeed confirmed that longer CPB duration was associated with a broader range of MACE, including ECMO needed, reoperation, and in-hospital mortality.

Additionally, a retrospective analysis of 3,889 patients reported that CPB duration in 10-minute increments was related to the risk of postoperative acute renal failure (OR = 1.06; 95% CI 1.01–1.12; *P* = 0.04) (23). Furthermore, an increase of 30 min in CPB duration was associated with hospital postoperative length of stay (*β* = 0.42; *P* < 0.0001), reoperation for bleeding (OR = 1.1; *P* = 0.017), multiorgan failure (OR = 1.21; *P* < 0.0001), and death after cardiac surgery (OR = 1.57; *P* < 0.0001) (11). Given the association between prolonged CPB duration and extended hospital stays, the present study emphasizes the necessity of closer postoperative monitoring for patients with longer CPB duration. This may include performing thorough assessments of vital signs, prioritizing surveillance of drainage output, and detecting physical examination changes that may require timely intervention (24).

Concerning the risk of re-thoracotomy for hemostasis, the incidence of bleeding after cardiac surgery ranged from 6.4% to 52.9% (24). In the first 6 h after cardiac surgery, every minute of duration of CPB increased bleeding by 0.003 ml/kg/h (24). Moreover, CPB duration was found to result in an approximately 50% decrease in platelet counts, which was associated with post-CPB blood loss (25). Although the specific impact of CPB duration on postoperative thoracotomy for hemostasis remains unclear, it is noteworthy that our study observed one case of postoperative re-exploration for hemostasis, with a CPB time of 154 min, which exceeded 120 min. Interventions aimed at minimizing hemodilution and replenishing coagulation factors may help reduce blood loss (25). For patients undergoing surgical repair of SVAS, restenosis was one of the main reasons for reoperation (4). Cardiac surgery with CPB could result in postoperative low cardiac output (26). Consequently, careful preoperative planning by an experienced surgeon—aiming to minimize CPB duration without compromising the quality of the repair—holds clinical significance for improving patients' outcomes.

In the present study, patients with CPB duration >2 h had higher likelihood of concomitant malformation, suggesting that patients with more severe stenosis could have longer CPB duration. Before PSM, patients with CPB duration >2 h were significantly older, heavier, and had lower EF. Diverse, a study of 5,006 patients found that the age at operation was negatively associated with CPB duration in a multiple linear regression (*β* = −0.45; 95% CI −0.6 to −0.3; *P* < 0.0001), while LVEF class (*β* = 5.85; 95% CI 2.8–8.9; *P* < 0.001) and the weight (*β* = 0.12; 95% CI 0–0.2; *P* = 0.03) were positively correlated to CPB duration in an univariate linear regression (11).

This study has benefited from some advantages. First, it has relatively large sample size, including patients from two different regions in China. Our analysis of a large multi-institutional cohort of patients who underwent surgical repair of SVAS during the last decade provides new insights related to the characteristics of patients and outcomes of SVAS surgical therapy in China. Second, PSM was regarded as the primary alternative for reducing confounding in observational studies (27). Moreover, various weighted multivariate logistic analyses were conducted to enhance the robustness of the results.

However, some limitations could not be ignored. Firstly, this was a retrospective study. Future well-designed cohort studies are needed to confirm this relationship. Second, we focused on CPB duration and its association with postoperative MACE, but did not include other predictors of adverse outcomes in this study. Although this study did not cover a broader range of cardiac surgeries, we speculated that the results could be generalized to other cardiac surgeries. Third, PSM could only adjust for known confounding factors. Although this study included as many preoperative variables as possible in the propensity score matching, some potential unknown confounding factors remain unaddressed. Additionally, all participants were Chinese. Although the study population is drawn from two distinct centers located in northern and southern China, future studies should further investigate whether these results are applicable across different races and regions.

## Conclusions

This study demonstrated a positively significant association between CPB duration and early postoperative MACE in patients undergoing surgical repair of SVAS, with an OR of 18.13 (95% CI 2.33–140.86; *P* = 0.006) in the PS-matching multivariate logistic regression model. It emphasized the importance of minimizing CPB duration for the prognosis of patients with SVAS. Patients with extended CPB duration warrant closer monitoring.

## Data Availability

The original contributions presented in the study are included in the article/Supplementary Material, further inquiries can be directed to the corresponding authors.
